# The Hallucinogenic Serotonin_2A_ Receptor Agonist, 2,5-Dimethoxy-4-Iodoamphetamine, Promotes cAMP Response Element Binding Protein-Dependent Gene Expression of Specific Plasticity-Associated Genes in the Rodent Neocortex

**DOI:** 10.3389/fnmol.2021.790213

**Published:** 2021-12-24

**Authors:** Lynette A. Desouza, Madhurima Benekareddy, Sashaina E. Fanibunda, Farhan Mohammad, Balaganesh Janakiraman, Utkarsha Ghai, Tamar Gur, Julie A. Blendy, Vidita A. Vaidya

**Affiliations:** ^1^Department of Biological Sciences, Tata Institute of Fundamental Research, Mumbai, India; ^2^Medical Research Centre, Kasturba Health Society, Mumbai, India; ^3^Department of Psychiatry and Behavioral Health, The Ohio State University College of Medicine, Columbus, OH, United States; ^4^Department of Systems Pharmacology and Translational Therapeutics, Perelman School of Medicine, University of Pennsylvania, Philadelphia, PA, United States

**Keywords:** 5-HT_2A_ receptor, cAMP response element binding protein, immediate early gene, BDNF, *Arc*, serotonergic psychedelic, cortical neuron, CREB deficient mice

## Abstract

Psychedelic compounds that target the 5-HT_2A_ receptor are reported to evoke psychoplastogenic effects, including enhanced dendritic arborization and synaptogenesis. Transcriptional regulation of neuronal plasticity-associated genes is implicated in the cytoarchitectural effects of serotonergic psychedelics, however, the transcription factors that drive this regulation are poorly elucidated. Here, we addressed the contribution of the transcription factor cyclic adenosine monophosphate (cAMP)-response element binding protein (CREB) in the regulation of neuronal plasticity-associated genes by the hallucinogenic 5-HT_2A_ receptor agonist, 2,5-dimethoxy-4-iodoamphetamine (DOI). *In vitro* studies with rat cortical neurons indicated that DOI enhances the phosphorylation of CREB (pCREB) through mitogen-activated protein (MAP) kinase and calcium/calmodulin dependent kinase II (CaMKII) pathways, with both cascades contributing to the DOI-evoked upregulation of *Arc, Bdnf1, Cebpb*, and *Egr2* expression, whilst the upregulation of *Egr1* and *cFos* mRNA involved the MAP kinase and CaMKII pathway respectively. We observed a robust DOI-evoked increase in the expression of several neuronal plasticity-associated genes in the rat neocortex *in vivo*. This DOI-evoked upregulation of neuronal plasticity-associated genes was completely blocked by the 5-HT_2A_ receptor antagonist MDL100,907 *in vitro* and was also abrogated in the neocortex of 5-HT_2A_ receptor deficient mice. Further, 5-HT_2A_ receptor stimulation enhanced pCREB enrichment at putative cAMP response element (CRE) binding sites in the *Arc*, *Bdnf1*, *Cebpb*, *cFos*, but not *Egr1* and *Egr2*, promoters in the rodent neocortex. The DOI-mediated transcriptional induction of *Arc*, *cFos* and *Cebpb* was significantly attenuated in the neocortex of CREB deficient/knockout (CREBαδ KO) mice. Collectively, these results indicate that the hallucinogenic 5-HT_2A_ receptor agonist DOI leads to a rapid transcriptional upregulation of several neuronal plasticity-associated genes, with a subset of them exhibiting a CREB-dependent regulation. Our findings raise the intriguing possibility that similar to slow-acting classical antidepressants, rapid-action serotonergic psychedelics that target the 5-HT_2A_ receptor may also recruit the transcription factor CREB to enhance the expression of neuronal plasticity-associated genes in the neocortex, which could in turn contribute to the rapid psychoplastogenic changes evoked by these compounds.

## Introduction

There has been a renewal of interest in serotonergic psychedelics as potential rapid-acting antidepressants for the treatment of anxiety and depression (Nutt et al., 2020; Vollenweider and Preller, 2020; Banks et al., 2021; De Gregorio et al., 2021). Most serotonergic psychedelics target the serotonin_2A_ (5-HT_2A_) receptor, and agonist action at the 5-HT_2A_ receptor is implicated in the molecular, cytoarchitectural, hallucinogenic and mood-related behavioral effects of serotonergic psychedelics (Vollenweider et al., 1998; González-Maeso et al., 2003, 2007; Martin and Nichols, 2016; López-Giménez and González-Maeso, 2018; Preller et al., 2018; Madsen et al., 2019; Slocum et al., 2021). Diverse serotonergic psychedelics, as well as the hallucinogenic 5-HT_2A_ receptor agonist, 2,5-dimethoxy-4-iodoamphetamine (DOI), can elicit both unique and distinctive molecular, behavioral and electrophysiological effects (Aghajanian and Marek, 1999; González-Maeso et al., 2003, 2007; Marek, 2017; Banerjee and Vaidya, 2020; Savino and Nichols, 2021). Common to all of these 5-HT_2A_ receptor agonists is a regulation of neuronal structural plasticity in the neocortex; from increased dendritic complexity to enhanced synaptogenesis (Ly et al., 2018, 2020; Savalia et al., 2021; Shao et al., 2021; Vargas et al., 2021). The rapid transcriptional effects evoked *via* agonistic action at the 5-HT_2A_ receptor, in particular the enhanced expression of neuronal plasticity-associated gene expression, are implicated in contributing to these psychoplastogenic effects (Nichols et al., 2003; Olson, 2018; Berthoux et al., 2019; Artin et al., 2021; de Vos et al., 2021; Jefsen et al., 2021).

5-HT_2A_ receptors are G-protein coupled receptors that drive Gq-signaling to activate phospholipase C beta (PLCβ) -mediated cleavage of phosphatidylinositol bisphosphate (PIP2) into inositol triphosphate (IP3) and diacylglycerol (DAG) (Raymond et al., 2001; Sharp and Barnes, 2020). IP3 mobilizes release of calcium from intracellular endoplasmic reticulum stores, which activates calcium/calmodulin dependent kinase II (CaMKII), while DAG activates protein kinase C (PKC) that phosphorylates and activates mitogen-activated protein (MAP) kinase (MAPK) signaling (Banerjee and Vaidya, 2020). These kinases in turn are reported to enhance the phosphorylation of the transcription factor, cyclic adenosine monophosphate (cAMP)-response-element binding protein (CREB), that has been previously shown to regulate the expression of several neuronal plasticity-associated genes (Lonze and Ginty, 2002; Sakamoto et al., 2011; Belgacem and Borodinsky, 2017). CREB is also a central target for diverse classes of antidepressant treatments, contributes to the neurotrophic, neurogenic and behavioral effects of antidepressants (Nibuya et al., 1996; Duman and Nibuya, 1997; Thome et al., 2000; Chen et al., 2001; Nair and Vaidya, 2006), and is dysregulated in both animal models of depression (Carlezon et al., 2005; Duman et al., 2005; Krishnan and Nestler, 2008, 2010) and in major depressive disorder patients (Blendy et al., 1996; Koch et al., 2009). Here, we sought to address whether CREB plays an important role in the regulation of neuronal plasticity-associated gene expression within the neocortex that arise in response to treatment with the hallucinogenic 5-HT_2A_ receptor agonist, DOI. Using both *in vitro* and *in vivo* approaches, as well as a CREB-deficient mouse line, we demonstrate that CREB contributes to the DOI-mediated regulation of a subset of neuronal plasticity-associated genes within the neocortex. This raises the intriguing possibility that similar to classical antidepressants, serotonergic psychedelics that target the 5-HT_2A_ receptor may also recruit CREB-mediated transcriptional regulation of specific neuronal plasticity-associated genes in the neocortex, thus contributing to the effects on neuronal structural plasticity and mood-related behavior.

## Materials and Methods

### Animal Treatments

Male Sprague-Dawley rats (2–3 months), serotonin_2A_ receptor knockout (5-HT_2A_^–/–^) mice (Weisstaub et al., 2006) and wild-type (WT) littermate controls (4–5 months) maintained on a 129S6/SvEv background were bred in the Tata Institute of Fundamental Research (TIFR) animal facility and CREB deficient/knockout (CREBαδ KO) mice were bred in the University of Pennsylvania animal facility and used for all experiments. For experiments using CREB deficient mice, the hypomorphic CREBαδ knockout mouse line that lacks the α and δ isoforms of CREB (CREBαδ KO) were used (Blendy et al., 1996). CREBαδ KO mice were generated as previously described (Walters and Blendy, 2001) and were maintained as F1 hybrids of 129SvEvTac:C57BL/6. For all experiments, CREBαδ KO mice and the WT controls were generated *via* crossing heterozygote CREBαδ 129SvEvTac with heterozygote CREBαδ C57BL/6 mice, allowing for a uniform genetic background in the experimental cohort. For the establishment of *in vitro* cortical cultures, rat embryos were derived at embryonic day 17.5 (E17.5) from timed pregnant Sprague-Dawley dams. All animal procedures using Sprague-Dawley rats and serotonin_2A_ receptor knockout (5-HT_2A_^–/–^) mice were carried out in accordance with the Committee for Care and Supervision of Experimental Animals (CPCSEA) and approved by the TIFR Institutional Animal Ethics Committee. All experiments with the CREBαδ KO mouse line were carried out in accordance with the NIH guideline for the care and use of laboratory animals and were approved by the University of Pennsylvania Animal Care and Use Committee. Animals were group housed and maintained on a 12 h light–dark cycle (lights on at 7 am) with access to food and water *ad libitum*.

Sprague-Dawley rats, CREBαδ KO and litter-matched WT mice, received intraperitoneal injections of the 5-HT_2A_ agonist, 2,5-dimethoxy-4-iodoamphetamine (DOI, 8 mg/kg, Sigma-Aldrich, United States) or vehicle (0.9% NaCl) (Vaidya et al., 1997). 5-HT_2A_^–/–^ and litter-matched WT mice received intraperitoneal administration of DOI (2 mg/kg) or vehicle (0.9% saline) and animals were sacrificed 2 h post drug administration (Vaidya et al., 1997; Benekareddy et al., 2012).

To make comparisons with a rapid-action antidepressant treatment, we also subjected a cohort of Sprague Dawley rats to electroconvulsive seizure (ECS) treatment *via* ear-clip electrodes (ECS unit, UGO Basile, Comerio, Italy) (frequency: 100 pulses/s; pulse width: 0.9 ms; pulse duration: 0.5 s; current: 80 mA). ECS animals were subjected to a single ECS treatment while sham-treated control animals underwent the application and removal of ear-clip electrodes without electrical stimulation, and were sacrificed 2 h post treatment. Animals were decapitated immediately, neocortex was dissected and snap frozen in liquid nitrogen.

### Cortical Neuron Cultures

Primary cortical neuron cultures were established from E17.5 rat embryos as described previously (Desouza et al., 2011; Fanibunda et al., 2019). Rat embryonic cortices were dissected and treated with trypsin-EDTA for 10 min, prior to dissociation in culture medium - Neurobasal medium supplemented with 2% B27 supplement, 0.5 mM L-glutamine, 5 U/ml penicillin and 5 U/ml streptomycin (Invitrogen, United States). Cells were plated on poly-D-lysine (Sigma-Aldrich, United States) coated 35 mm dishes at a density of 10^6^ cells/dish. Following attachment and neurite extension *in vitro* for a period of 7 days, neurons were treated with DOI (10 μM) or vehicle (DMSO) for 2 h on day *in vitro* (DIV) 10. In experiments to delineate signaling events downstream of 5-HT_2A_ receptor activation, neurons were treated with DOI (10 μM) for 2 h in the presence of the 5-HT_2A_ receptor antagonist, MDL100,907 (10 μM) and specific signaling pathway inhibitors, namely the phospholipase C (PLC) inhibitor U73122 (5 μM), the mitogen activated protein kinase kinase (MAPKK) inhibitor U0126 (50 μM) and the CaM kinase II (CaMKII) inhibitor KN-62 (10 μM) (Tocris Bioscience, United Kingdom). The inhibitors were added to the cultures 30 min prior to DOI treatment, and were present throughout the duration of DOI exposure. Following treatments, cortical neurons were processed for immunofluorescence, RNA extraction for qPCR analysis, or western blot analysis.

### Immunofluorescence

Immunofluorescence staining was performed as described previously (Fanibunda et al., 2019). In brief, cortical neurons were fixed in 4% paraformaldehyde, followed by blocking in 10% horse serum and incubated with primary antibodies, rabbit anti-pCREB (1:1000; Cell Signaling Technology, MA, United States) or goat anti-5-HT_2A_ receptor (1:500, Santa Cruz Biotechnologies, United States) along with the pan-neuronal marker, mouse anti-MAP2 (1:1000, Sigma-Aldrich, United States) overnight at 4°C. This was followed by incubation with secondary antibodies, Alexa 488 conjugated anti-goat (1:500; Molecular probes, CA, United States) or Alexa 488 conjugated anti-rabbit (1:500; Molecular Probes, CA, United States) or biotinylated horse anti-mouse (1:500, Roche Applied Science, Switzerland) with subsequent incubation with streptavidin-conjugated Alexa 568 (1:500, Molecular Probes, CA, United States) for 2 h. Following secondary antibody incubation and serial washes, cortical neurons were mounted in Vectashield (Vector Laboratories, CA, United States), and images were captured on the Zeiss LSM5 Exciter laser scanning microscope.

### Western Blot Analysis

Rat cortical neurons were lysed in Laemmli sample buffer (2% SDS, 10% glycerol, 60 mM Tris-Cl, 0.01% bromophenol blue) and proteins were resolved *via* sodium dodecyl sulfate polyacrylamide gel electrophoresis (SDS-PAGE), followed by transfer onto polyvinylidene fluoride (PVDF, GE Healthcare, United Kingdom) membranes. Blots were blocked in 5% fat-free milk in 0.05% Tris Buffered Saline-Tween 20 (TBS-T) and incubated in primary antibodies in 0.05% TBS-T, overnight at 4°C. Primary antibodies included rabbit anti-pCREB (1:500; Cell Signaling Technology, MA, United States) and rabbit anti-CREB (1:1000, Cell Signaling Technology). Blots were washed 3–5 times and incubated with a 1:5000 dilution of horseradish peroxidase-conjugated goat anti-rabbit antibody (GE Healthcare, United Kingdom) for 1 h. Protein-antibody complexes were detected on X-ray films following addition of Enhanced Chemiluminescence (ECL) substrate (GE Healthcare, United Kingdom). The relative density of the pCREB and CREB bands was quantitated using ImageJ software (NIH, United States), and was represented as a pCREB/CREB ratio.

### Quantitative PCR Analysis

RNA was isolated using Tri Reagent (Sigma-Aldrich, United States), according to the manufacturer’s protocols. Two μg of RNA per sample was reverse transcribed using a complementary DNA (cDNA) synthesis kit (QuantiTect reverse transcription kit, Qiagen, Germany). Quantitative PCR (qPCR) was performed in a Mastercycler^®^ ep realplex real-time PCR system (Eppendorf, Germany). cDNA was amplified using a SYBR Green kit (Applied Biosystems, CA, United States), with primers for the genes of interest (Supplementary Table 1). Hypoxanthine phosphoribosyl transferase (*Hprt*) was used as an endogenous housekeeping gene control for normalization. The relative expression levels between control and treated samples were computed by the comparative Ct method, as described previously (Schmittgen and Livak, 2008). Data are represented as fold change ± SEM as compared to control.

### Chromatin Immunoprecipitation

Chromatin immunoprecipitation (ChIP) was carried out as described previously (Desouza et al., 2011). Briefly, bilateral frontal neocortices were dissected and fixed to crosslink the DNA with the bound proteins. The tissue was placed in a pre-chilled Dounce homogenizer, sonicated and immunoprecipitated using a phosphorylated CREB (pCREB) antibody (1 μg; Cell Signaling Technology, MA, United States). Following reverse crosslinking and chromatin precipitation, qPCR analysis was performed for upstream sequences of the *Arc*, *BdnfI*, *Cebpb*, *cFos*, *Egr1*, and *Egr2* promoters that contained putative CRE sites as predicted using AliBaba 2.1^1^. For each sample the results were normalized to the input chromatin from the same sample. (Primer sequences used in ChIP experiments: Supplementary Table 2).

### Dimethoxy-4-Iodoamphetamine-Mediated Head Twitch Behavior

Administration of DOI results in a stereotypical head twitch behavior, characterized by rapid radial movements of the head (Canal and Morgan, 2012; Halberstadt and Geyer, 2013). This behavior was videotaped in the home cage for a total duration of 20 min, commencing 20 min following administration of the DOI or vehicle (saline) treatment. The total number of head twitches in this time window were counted by an experimenter blind to the experimental treatment groups.

### *In situ* Hybridization

CREBαδ KO and litter-matched WT mice were administered DOI and vehicle (Veh) resulting in four groups: WT + Veh, WT + DOI, CREBαδ KO + Veh, CREBαδ KO + DOI. Mice were anesthetized with sodium thiopentone and transcardially perfused with 4% paraformaldehyde (PFA). The brains were postfixed in 4% PFA overnight and cryoprotected in 30% sucrose in 4% PFA prior to being shipped to TIFR, India. Coronal sections of 30 μM thickness were cut on the freezing microtome (Leica Biosystems, Germany), fixed, blocked and acetylated. The floating sections were incubated for 20 h at 60°C in a hybridization buffer (50% formamide, 1xSSC, 25xDenhardt’s solution, 40 mM dithiothreitol, 150 μg/ml yeast tRNA, 10% dextran sulfate, 400 μg/ml salmon sperm DNA) containing ^35^S-UTP labeled antisense riboprobes for *Arc* mRNA at a concentration of 1 × 10^6^ cpm/300 μl. Antisense riboprobes to *Arc* mRNA were generated from a transcription-competent plasmid kindly provided by Dr. Oswald Steward (University of California, Irvine, CA, United States). Following hybridization, all sections were washed in ribonuclease A (20 mg/ml; USB corporation, United States), followed by stringent washes in decreasing concentrations of SSC, mounted on slides, air dried and exposed to Hyper film β-max (GE Healthcare, United States) for 7 days. Levels of Arc mRNA were quantified using Scion Image (Scion, United States) and calibrated using ^14^C standards to correct for non-linearity. Equivalent areas of the somatosensory and prefrontal cortex were outlined and optical density measurements were determined (3–4 sections/animal).

### Statistical Analysis

Results were subjected to statistical analysis using Student’s unpaired *t*-test for experiments with two groups (GraphPad InStat) and one-way ANOVA (GraphPad, Prism 8) for experiments using signaling pathway inhibitors, followed by Tukey’s *post hoc* test for group comparisons. For four group experiments statistical analysis was performed using two-way ANOVA (GraphPad, Prism 8). Tukey’s *post hoc* test for group comparisons was applied only when there was a significant two-way ANOVA interaction observed between the two variables of DOI treatment and 5-HT_2A_^–/–^ KO genotype or DOI treatment and CREBαδ KO genotype. Statistical significance was determined at *p* < 0.05.

## Results

### Acute Treatment With the 5-HT_2A_ Receptor Agonist, Dimethoxy-4-Iodoamphetamine, Regulates Neuronal Plasticity-Associated Gene Expression *via* the Mitogen-Activated Protein Kinase and CaMKII Signaling Pathways and Enhances Phosphorylated cAMP Response Element Binding Protein Expression *in vitro*

**FIGURE 1:**
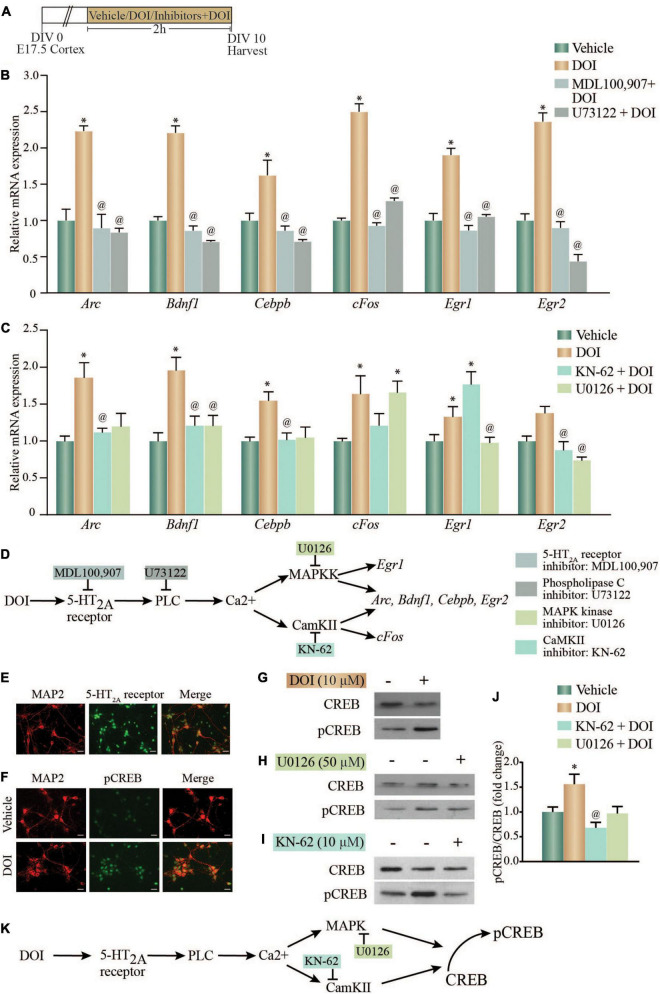
Acute treatment with the 5-HT_2A_ receptor agonist, DOI regulates neuronal plasticity-associated gene expression *via* the MAP kinase and CaMKII signaling pathways and enhances phosphorylated CREB (pCREB) expression *in vitro.* **(A)** Shown is a schematic of the treatment paradigm for cortical neurons derived from E17.5 rat embryos, allowed to differentiate till day *in vitro* (DIV) 10, following which neurons were treated with vehicle (DMSO) or the 5-HT_2A_ receptor agonist, DOI (10 μM), in the presence or absence of CaMKII and MAP kinase signaling pathway inhibitors (CaMKII inhibitor: KN-62; MAPKK inhibitor: U0126). **(B)** Shown is the relative mRNA expression for plasticity-associated genes following DOI treatment in the presence or absence of the 5-HT_2A_ receptor antagonist, MDL100,907 or PLC inhibitor, U73122, represented as fold change of vehicle ± SEM. (Representative results from *n* = 4 wells per treatment group/*N* = 2, *p < 0.05 as compared to vehicle, ^@^*p* < 0.05 as compared to DOI, one-way ANOVA, Tukey’s *post hoc* test). **(C)** Shown is the relative mRNA expression for plasticity-associated genes following DOI treatment in the presence or absence of MAP kinase and CaMKII signaling pathway inhibitors, represented as fold change of vehicle ± SEM. (Representative results from *n* = 3–5 wells per treatment group/*N* = 3, *p < 0.05 as compared to vehicle, ^@^*p* < 0.05 as compared to DOI, one-way ANOVA, Tukey’s *post hoc* test). **(D)** Shown is a schematic summarizing the putative signaling pathways that may contribute to DOI-induced gene expression. The CaMKII inhibitor, KN-62 and the MAPKK inhibitor, U0126 inhibit the CaMKII and MAP kinase signaling pathways respectively. The DOI-mediated upregulation of *Arc*, *Bdnf1*, *Cebpb*, and *Egr2* mRNA levels was blocked by both the MAPKK and CaMKII inhibitors, whereas the increase in *cFos* mRNA was blocked by the CaMKII, not the MAPKK, inhibitor and the upregulation of *Egr1* mRNA was blocked by the MAPKK, not the CaMKII, inhibitor. **(E)** Shown are representative immunofluorescence images of rat cortical neurons *in vitro* with double staining for the neuronal marker MAP2 (red) and 5-HT_2A_ receptor (green). Scale bar: 30 μm. Magnification: 20X. **(F)** Shown are representative immunofluorescence images of rat cortical neurons with double staining for pCREB (green) and the neuronal marker MAP2 (red) – upper panel: Vehicle; lower panel: DOI. Scale bar: 30 μm. Magnification: 20X. **(G–J)** Shown are representative immunoblots for pCREB and CREB protein levels in rat cortical neurons treated with DOI **(G)** or with DOI in the presence or absence of the MAPKK inhibitor U0126 **(H)** or the CaMKII inhibitor KN-62 **(I)**. **(J)** Quantitative densitometric analysis of pCREB/CREB levels in rat cortical neurons treated with DOI in the presence or absence of U0126 or KN-62. Results are expressed as fold change of vehicle ± SEM. (Representative results from *n* = 3–5 wells per treatment group/*N* = 3, *p < 0.05 as compared to vehicle, ^@^*p* < 0.05 as compared to DOI, one-way ANOVA, Tukey’s *post hoc* test). **(K)** Shown is a schematic depicting the putative pathway *via* which pCREB levels are enhanced following DOI administration, indicative of a role for the MAP kinase and CaMKII signaling pathways.

The 5-HT_2A_ receptor agonist DOI, is a potent hallucinogen that is known to evoke an increase in the expression of several neuronal plasticity-associated genes (González-Maeso et al., 2003). Following ligand binding, the Gq-coupled 5-HT_2A_ receptor can differentially recruit multiple signaling pathways to bring about distinct signaling responses. We sought to address the contribution of the 5-HT_2A_ receptor, the Gq-coupled phospholipase C (PLC) signaling pathway, the MAP kinase and CaM Kinase II (CaMKII) signaling pathways and the transcription factor CREB to the DOI-evoked induction of neuronal plasticity-associated gene expression. We stimulated primary rat cortical neurons *in vitro* with DOI, in the presence or absence of the 5-HT_2A_ receptor antagonist, MDL100,907; PLC inhibitor, U73122; CaMKII inhibitor, KN-62 or the MAPKK inhibitor, U0126 (Figure 1A). We observed an increase in transcript expression of *Arc*, *Bdnf1*, *Cebpb, cFos, Egr1*, and *Egr2* mRNA levels following DOI treatment, which was inhibited by the 5-HT_2A_ receptor antagonist MDL100,907, as well as the PLC signaling pathway inhibitor, U73122 (Figure 1B) {one-way ANOVA: *Arc*: [*F*_(3_,_12)_ = 11.23, *p* = 0.0008]; *Bdnf1*: [*F*_(3_,_12)_ = 90.59, *p* < 0.0001]; *Cebpb*: [*F*_(3_,_12)_ = 14.78, *p* = 0.0002], *cFos*: [*F*_(3_,_12)_ = 135.6, *p* < 0.0001], *Egr1*: [*F*_(3_,_12)_ = 26.17, *p* < 0.0001], *Egr2*: [*F*_(3_,_12)_ = 28.45, *p* < 0.0001]}. These observations indicate that the 5-HT_2A_ receptor mediates the DOI-evoked induction in neuronal plasticity-associated genes, *via* recruiting the Gq-coupled PLC pathway. We next sought to delineate the contribution of the downstream MAP kinase and CaM Kinase II (CaMKII) signaling pathways to the DOI-evoked upregulation of neuronal plasticity-associated genes. We observed an upregulation of *Arc*, *Bdnf1*, *Cebpb*, and *Egr2* mRNA levels following DOI treatment, which was inhibited by both the CaMKII inhibitor KN-62 and the MAPKK inhibitor U0126 (Figure 1C) {one-way ANOVA: *Arc*: [*F*_(3_,_9)_ = 7.551, *p* = 0.008]; *Bdnf1*: [*F*_(3_,_11)_ = 6.436, *p* = 0.009]; *Cebpb*: [*F*_(3_,_8)_ = 5.293, *p* = 0.027], *Egr2*: [*F*_(3_,_10)_ = 7.685, *p* = 0.006]}. In contrast, the DOI-mediated upregulation of *cFos* mRNA levels was prevented by only the CaMKII inhibitor KN-62, while the increase in the *Egr1* transcript levels was abrogated exclusively by the MAPKK inhibitor U0126 (Figure 1C) {one-way ANOVA: *cFos*: [*F*_(3_,_11)_ = 5.563, *p* = 0.014], *Egr1*: [*F*_(3_,_11)_ = 14.02, *p* = 0.0004]}. This indicates a differential involvement of the MAP kinase and the CaMKII pathway in the 5-HT_2A_ receptor-mediated transcriptional regulation of specific neuronal plasticity-associated genes (Figure 1D).

We next sought to address the possible role of the transcription factor CREB in mediating the signaling events evoked by DOI, downstream of the 5-HT_2A_ receptor. The DOI regulated genes, *Arc*, *Bdnf1*, *Cebpb*, *cFos*, *Egr1*, and *Egr2* were found to contain putative cAMP response element (CRE) (TGACG/CGTCA/TGACGTCA) sites in the upstream promoter regions, suggestive of the possibility of a CREB-dependent transcriptional regulation. We first confirmed 5-HT_2A_ receptor expression in cortical neurons, by immunofluorescence staining (Figure 1E). Immunostaining with the phosphorylated CREB (pCREB) antibody demonstrated an increase in pCREB immunofluorescence intensity in DOI-treated rat cortical neurons as compared to vehicle-treated control neurons (Figure 1F). Immunoblotting to detect pCREB levels, also demonstrated a robust increase in pCREB/CREB levels in DOI-treated cortical neurons (Figure 1G), further corroborating that DOI treatment enhances pCREB levels. Further, the MAPKK inhibitor (Figure 1H) and the CaMKII inhibitor (Figure 1I), both prevented the DOI-evoked increase in pCREB/CREB levels, demonstrating a role for the MAP kinase pathway and CaMKII pathway in mediating the DOI-evoked increase in pCREB levels (Figure 1J) [pCREB/CREB: *F*_(3_,_17)_ = 5.561, *p* = 0.008]. This data collectively suggests that DOI, a hallucinogenic ligand of the Gq-coupled 5-HT_2A_ receptor, recruits the PLC pathway driving MAP kinase and CaMKII signaling to enhance the phosphorylation of the transcription factor CREB in rat cortical neurons (Figure 1K).

### Dimethoxy-4-Iodoamphetamine-Mediated Regulation of Plasticity-Associated Gene Expression in the Neocortex Is Altered in 5-HT_2A_^–/–^ Receptor Deficient Mice

**FIGURE 2:**
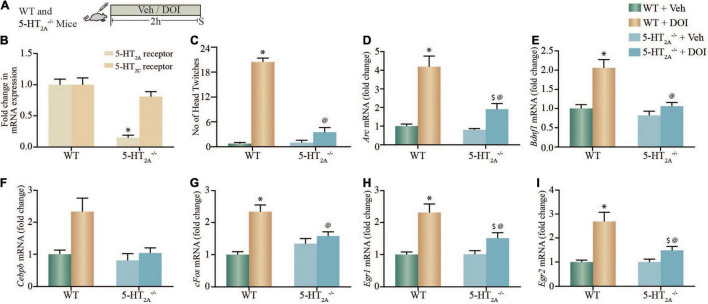
DOI-mediated regulation of plasticity-associated gene expression in cortical brain regions is altered in serotonin_2A_ receptor knockout (5-HT_2A_^–/–^) mice. **(A)** Shown is a schematic of the acute treatment paradigm for wild type (WT) and 5-HT_2A_ receptor deficient (5-HT_2A_^–/–^) mice, with vehicle (saline) or DOI (2 mg/kg) followed by sacrifice 2 h after treatment. **(B)** The bar graph depicts quantitative qPCR analysis for 5-HT_2A_ and 5-HT_2C_ receptor mRNA in the neocortex of WT and 5-HT_2A_^–/–^ mice represented as fold change of WT ± SEM. (*n* = 12 animals per treatment group, *p < 0.05 as compared to WT, unpaired Students *t*-test). **(C)** The bar graph depicts the quantitation of head-twitch responses evoked in response to acute treatment with the 5-HT_2A_ receptor agonist, DOI or vehicle in both WT and 5-HT_2A_^–/–^ mice (*n* = 7–9 animals per treatment group, *p < 0.05 as compared to WT mice, ^@^*p* < 0.05 as compared to WT + DOI mice, two-way ANOVA, Tukey’s *post hoc* test). **(D–I)** Bar graphs depict quantitation of qPCR analysis for mRNA expression of *Arc* **(D)**, *Bdnf1* **(E)**, *Cebpb* **(F)**, *cFos* **(G)**, *Egr1* **(H)**, and *Egr2* **(I)**, following acute DOI or vehicle treatment to WT and 5-HT_2A_^–/–^ mice. (*n* = 12 animals per group, *p < 0.05 as compared to WT mice, ^$^*p* < 0.05 as compared to 5-HT_2A_^–/–^ mice, ^@^*p* < 0.05 as compared to WT + DOI mice, two-way ANOVA, Tukey’s *post hoc* test).

Given that the hallucinogen DOI, in addition to exhibiting agonist action at the 5-HT_2A_ receptor, also binds to the 5-HT_2*C*_ receptor with lower affinity (Titeler et al., 1988), we further sought to evaluate the contribution of the 5-HT_2A_ receptor to the DOI-mediated induction of neuronal plasticity-associated genes in 5-HT_2A_^–/–^ receptor deficient mice (Figure 2A). We confirmed that 5-HT_2A_^–/–^ mice exhibit both a robust reduction in cortical 5-HT_2A_, but not in 5-HT_2*C*_, mRNA expression (Figure 2B), accompanied by a significant decline in a stereotypical head twitch response (HTR) behavior evoked by the 5-HT_2A_ receptor agonist DOI (Canal and Morgan, 2012; Halberstadt and Geyer, 2013). Two-way ANOVA analysis for HTR events (Figure 2C) indicated a significant DOI by 5-HT_2A_^–/–^ genotype interaction [*F*_(1_,_29)_ = 113.4, *p* < 0.0001], as well as significant main effects of DOI [*F*_(1_,_29)_ = 189.1, *p* < 0.0001] and 5-HT_2A_^–/–^ genotype [F_(1_,_29)_ = 107.6, *p* < 0.0001].

We next performed qPCR analysis to assess whether the DOI-evoked upregulation of specific neuronal plasticity-associated genes *Arc, Bdnf1*, *Cebpb*, *cFos, Egr1*, and *Egr2* mRNA expression observed *in vitro*, was altered in the neocortex of WT and 5-HT_2A_^–/–^ mice following acute DOI administration (Figures 2D–I). We noted significant two-way ANOVA interactions of DOI by 5-HT_2A_^–/–^ genotype for the neocortical mRNA expression of *Arc* [*F*_(1_,_44)_ = 4.920, *p* = 0.0318], *Bdnf1* [*F*_(1_,_44)_ = 4.45, *p* = 0.04], *cFos* [*F*_(1_,_44)_ = 12.59, *p* = 0.0009], *Egr1* [*F*_(1_,_44)_ = 4.257, *p* = 0.045] and *Egr2* [*F*_(1_,_44)_ = 1118, *p* < 0.0001], but not for *Cebpb* mRNA expression [*F*_(1_,_44)_ = 2.49, *p* = 0.12]. We also noted significant main effects of DOI for *Arc* [*F*_(1_,_44)_ = 81.38, *p* < 0.0001], *Bdnf1* [*F*_(1_,_44)_ = 20.37, *p* < 0.0001], *Cebpb* [*F*_(1_,_44)_ = 8.518, *p* = 0.0055], *cFos* [*F*_(1_,_44)_ = 26.72, *p* < 0.0001], *Egr1* [*F*_(1_,_44)_ = 34.58, *p* < 0.0001], and *Egr2* [*F*_(1_,_44)_ = 1392, *p* < 0.0001] mRNA expression in the neocortex. Significant main effects of 5-HT_2A_^–/–^ genotype were observed for neocortical mRNA levels of *Arc* [*F*_(1_,_44)_ = 15.79, *p* = 0.0003], *Bdnf1* [*F*_(1_,_44)_ = 15.77, *p* = 0.0003], *Cebpb* [*F*_(1_,_44)_ = 7.399, *p* = 0.009], and *Egr2* [*F*_(1_,_44)_ = 1122, *p* < 0.0001], but not for *cFos* and *Egr1* mRNA expression. *Post hoc* Tukey comparison analysis revealed that the DOI-evoked upregulation of *Bdnf1* and *cFos* expression noted in the neocortex of WT mice was completely abrogated in 5-HT_2A_^–/–^ receptor deficient mice, and the DOI-evoked increase in *Arc*, *Egr1*, and *Egr2* neocortical mRNA levels noted in WT mice was significantly attenuated in the 5-HT_2A_^–/–^ receptor deficient genotype. Collectively, studies with pharmacological blockade of the 5-HT_2A_ receptor *in vitro*, and using 5-HT_2A_^–/–^ mice *in vivo*, indicate the critical contribution of the 5-HT_2A_ receptor to the DOI-mediated regulation of a subset of neuronal plasticity-associated genes in the neocortex.

### Acute Treatment With Dimethoxy-4-Iodoamphetamine Enhances Both the Expression of Putative cAMP Response Element-Containing Plasticity-Associated Genes and the Enrichment of Phosphorylation of cAMP Response Element Binding Protein Within the Promoter Regions of Specific Plasticity-Associated Genes in the Neocortex of Adult Rats

**FIGURE 3:**
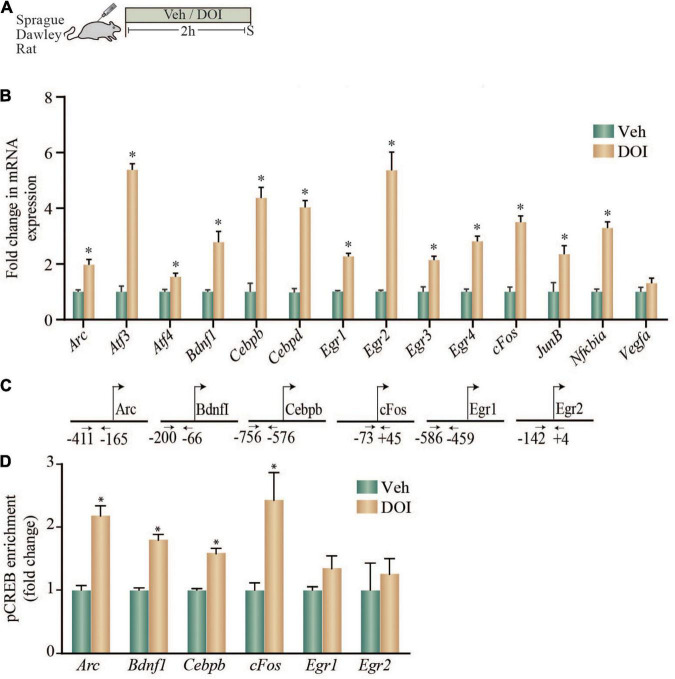
Acute treatment with DOI enhances both the expression of putative CRE-containing plasticity-associated genes and the enrichment of pCREB within the promoter regions of specific plasticity-associated genes in the neocortex of adult rats. **(A)** Shown is a schematic of the treatment paradigm wherein adult Sprague-Dawley rats were injected with vehicle or DOI (8 mg/kg) and were sacrificed 2 h following treatment. **(B)** The bar graph indicates the fold change in mRNA expression of specific plasticity-associated genes in the neocortex of vehicle and DOI-treated rats represented as fold change of vehicle ± SEM (*n* = 4–6 per treatment group, *p < 0.05 as compared to vehicle, unpaired Students *t*-test). **(C)** Shown are the chromatin immunoprecipitation (ChIP) PCR amplicons with primer locations spanning putative CRE sequences in the upstream gene regulatory sequences for *Arc*, *Bdnf1*, *Cebpb*, *cFos*, *Egr1*, and *Egr2*. **(D)** Shown is a bar graph for pCREB enrichment at the *Arc*, *Bdnf1*, *Cebpb*, *cFos*, *Egr1*, and *Egr2* promoters based on ChIP analysis performed on tissue derived from the neocortex of vehicle and DOI treated adult rats. Results are expressed as the fold change of vehicle ± SEM. (*n* = 7–10 animals per treatment group, *p < 0.05 as compared to vehicle, unpaired Students *t*-test).

Given that we observed that the 5-HT_2A_ receptor agonist, DOI, induced a robust upregulation of neuronal plasticity-associated genes in rat cortical neurons in culture, as well as in WT but not 5-HT_2A_^–/–^ mice, we next examined whether DOI administration to Sprague-Dawley male rats could evoke similar alterations in neuronal plasticity-associated genes in the neocortex. We systemically administered DOI (8 mg/kg) and evaluated gene expression in the neocortex at a 2 h time-point post treatment (Figure 3A). DOI administration evoked a robust increase in the gene expression of *Arc*, *Atf3*, *Atf4*, *Bdnf1*, *Cebpb*, *Cebpd*, *Egr1*, *Egr2*, *Egr3*, *Egr4*, *cFos*, *JunB*, and *Nfkbia* in the rat neocortex (Figure 3B).

We next examined if a subset of the genes upregulated by DOI treatment, that are known to contain putative CRE binding sites in their promoter regions based on *in silico* analysis, also exhibit enrichment for pCREB within their promoters (Figure 3C). Enhanced expression of *Arc*, *Bdnf1*, *Cebpb*, and *cFos* in the neocortex of DOI-treated animals was accompanied by a significant enrichment of pCREB at the promoters of *Arc*, *Bdnf1*, *Cebpb*, and *cFos* (Figure 3D). In contrast, the enhanced gene expression of *Egr1* and *Egr2* was not associated with any significant change in pCREB enrichment at putative CRE sites within their promoter regions (Figure 3D). Taken together, these results indicate that *in vivo* administration of the 5-HT_2A_ receptor agonist DOI, evokes a robust upregulation of several neuronal plasticity-associated genes in the neocortex, a subset of which exhibit significant pCREB enrichment in their promoter regions.

To draw a comparison of the nature and magnitude of regulation of this subset of neuronal plasticity-associated genes in the neocortex by the serotonergic hallucinogen, DOI, with that evoked by a rapid-action antidepressant treatment, electroconvulsive seizure (ECS) treatment, neocortices derived from acute ECS or sham treated rats were subjected to qPCR analysis (Supplementary Figure 1). We noted a robust increase in *Arc, Bdnf1, Cebpb, cFos*, and *Egr2* but not *Egr1* mRNA levels following acute ECS treatment (Supplementary Figure 1), with the nature and scale of upregulation comparable to that following DOI treatment.

### Dimethoxy-4-Iodoamphetamine-Mediated Regulation of Plasticity-Associated Gene Expression in Cortical Brain Regions Is Perturbed in CREBαδ Knockout (CREBαδ KO) Mice

**FIGURE 4:**
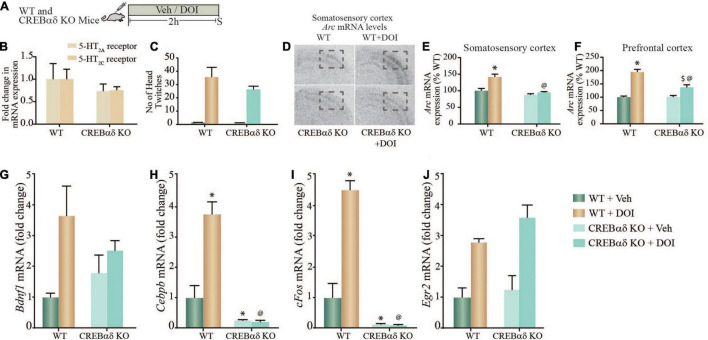
DOI-mediated regulation of plasticity-associated gene expression in cortical brain regions is perturbed in CREBαδ knockout (CREBαδ KO) mice. **(A)** Shown is a schematic of the acute treatment paradigm for wild type (WT) and CREBαδ KO mice, with vehicle (saline) or DOI (8 mg/kg) followed by sacrifice 2 h after treatment. **(B)** The bar graph depicts quantitative qPCR analysis for 5-HT_2A_ and 5-HT_2C_ receptors in the neocortex of WT and CREBαδ KO mice represented as fold change of WT ± SEM. (*n* = 3–4 animals per treatment group). **(C)** The bar graph depicts the quantitation of head-twitch responses evoked in response to acute treatment with the 5-HT_2A_ receptor agonist, DOI or vehicle in both WT and CREBαδ KO mice (*n* = 4 animals per treatment group). **(D)** Shown are representative autoradiographs for *Arc* mRNA expression in the neocortex from WT, WT + DOI, CREBαδ KO, and CREBαδ KO + DOI mice, with the outline inset indicating the somatosensory region. Shown are bar graphs for the quantitative densitometric analysis of levels of *Arc* mRNA expression in the somatosensory **(E)** and prefrontal **(F)** cortex following DOI or vehicle treatment to WT and CREBαδ KO mice. Results are represented as percentage of WT and are mean ± SEM (*n* = 3–4 animals per group, *p < 0.05 as compared to WT + Veh mice, ^$^*p* < 0.05 as compared to CREBαδ KO + Veh mice, ^@^*p* < 0.05 as compared to WT + DOI mice, two-way ANOVA, Tukey’s *post hoc* test). **(G–J)** Bar graphs depict quantitation of qPCR analysis for mRNA expression of *Bdnf1* **(G)**, *Cebpb* **(H)**, *cFos* **(I)**, and *Egr2* **(J)**, following acute DOI or vehicle treatment to WT and CREBαδ KO mice. (*n* = 3–4 animals per group, *p < 0.05 as compared to WT mice, ^$^*p* < 0.05 as compared to CREBαδ KO mice, ^@^*p* < 0.05 as compared to WT + DOI mice, two-way ANOVA, Tukey’s *post hoc* test).

Given the evidence both *in vitro* and *in vivo*, that the 5-HT_2A_ receptor agonist, DOI induces (1) a robust increase in pCREB levels in rat cortical neurons, (2) a significant enrichment of pCREB at the promoters of specific plasticity-associated genes that are robustly enhanced by DOI treatment, we next sought to address the contribution of CREB to the regulation of these transcripts. We used hypomorphic CREBαδ KO mice (Blendy et al., 1996) that are reported to have a greater than 90% reduction in CREB binding activity to consensus CRE target sites (Walters and Blendy, 2001). We treated WT and CREBαδ KO mice with vehicle or DOI (8 mg/kg), and assessed transcript expression of specific neuronal plasticity-associated genes at a 2 h time-point post treatment (Figure 4A). To rule out the possibility that CREB may indirectly regulate 5-HT_2A_ or 5-HT_2*C*_ receptors, we first assessed whether the baseline expression of these receptors was altered in CREBαδ KO mice as compared to their WT controls, and observed no change in 5-HT_2A_ or 5-HT_2*C*_ receptor mRNA levels within the neocortex (Figure 4B). Further to assess whether CREB deficient mice exhibit any change in 5-HT_2A_ receptor evoked behavioral responses, we evaluated the HTR behavior evoked by DOI. Behavioral analysis to quantify the number of HTR events in WT and CREBαδ KO mice, indicated that the number of HTR responses evoked by DOI were unaltered in CREBαδ KO mice (Figure 4C). While we noted no significant two-way ANOVA interaction of DOI and CREBαδ KO genotype, we observed a significant main effect of DOI [*F*_(1_,_12)_ = 58.64, *p* < 0.0001]. This indicates that the loss of CREBαδ isoforms does not alter either the expression of the 5-HT_2A_ receptor, or the behavioral HTRs evoked by the 5-HT_2A_ receptor agonist DOI, that are critically dependent on the cortical 5-HT_2A_ receptor.

We next performed *in situ* hybridization and qPCR analysis to address whether the regulation of specific neuronal plasticity-associated genes that exhibit pCREB enrichment at their promoters following DOI treatment, were altered in the neocortex of CREBαδ KO mice. Radioactive *in situ* hybridization analysis indicated that the DOI-evoked robust induction of *Arc* mRNA levels both in the somatosensory cortex and in the prefrontal cortex was significantly attenuated in CREBαδ KO mice (Figure 4D). Two-way ANOVA analysis for *Arc* mRNA levels in the somatosensory cortex (Figure 4E) indicated a significant DOI by CREBαδ KO genotype interaction [*F*_(1_,_12)_ = 8.39, *p* = 0.013], as well as significant main effects of DOI [*F*_(1_,_12)_ = 16.81, *p* = 0.002] and CREBαδ KO genotype [*F*_(1_,_12_) = 24.39, *p* = 0.0003]. The robust DOI-evoked upregulation of *Arc* mRNA in the somatosensory cortex of WT control mice was completely lost in the CREBαδ KO mice. Two-way ANOVA analysis for *Arc* mRNA levels in the prefrontal cortex (Figure 4F) indicated a significant DOI by CREBαδ KO genotype interaction [*F*_(1_,_11)_ = 15.73, *p* = 0.002], as well as significant main effects of DOI [*F*_(1_,_11)_ = 78.83, *p* = 0.0001] and CREBαδ KO genotype [*F*_(1_,_11)_ = 14.75, *p* = 0.003]. The robust DOI-evoked upregulation of *Arc* mRNA in the prefrontal cortex of WT control mice was significantly attenuated in the CREBαδ KO mice. No change was observed in the basal expression of *Arc* mRNA in either the somatosensory or prefrontal of CREBαδ KO mice, which did not differ from vehicle-treated WT controls.

qPCR analysis was carried out to assess whether the DOI-evoked upregulation of *Bdnf1*, *Cebpb*, *cFos*, and *Egr2* mRNA expression was altered in the neocortex of CREBαδ KO mice following acute DOI administration (Figures 4G–J). Two-way ANOVA analysis for *Bdnf1* mRNA levels in the neocortex indicated no significant DOI by CREBαδ KO genotype interaction (Figure 4G), however we did observe a significant main effect of DOI [*F*_(1_,_10)_ = 10.19, *p* = 0.01] and no main effect of CREBαδ KO genotype. Two-way ANOVA analysis for *Cebpb* mRNA levels in the neocortex (Figure 4H) indicated a significant DOI by CREBαδ KO genotype interaction [*F*_(1_,_12)_ = 6.413, *p* = 0.026], as well as a trend toward a main effect of DOI [*F*_(1_,_12)_ = 3.388, *p* = 0.09] and a significant main effect of CREBαδ KO genotype [*F*_(1_,_12)_ = 50.49, *p* = 0.0001]. Tukey’s *post hoc* group comparisons indicated that the CREBαδ KO mice exhibited a significant baseline decrease (*p* = 0.03) in *Cebpb* mRNA levels in the neocortex. Further, while DOI-evoked a robust and significant upregulation of *Cebpb* mRNA levels in WT animals (*p* = 0.04), this was completely lost in the CREBαδ KO mice. *Cebpb* mRNA levels in the DOI-treated WT cohort differed significantly from the DOI-treated CREBαδ KO mice (*p* < 0.0001). Two-way ANOVA analysis for *cFos* mRNA levels in the neocortex (Figure 4I) indicated a significant DOI by CREBαδ KO genotype interaction [*F*_(1_,_12)_ = 8.966, *p* = 0.011], as well as a trend toward a main effect of DOI [*F*_(1_,_12)_ = 4.493, *p* = 0.056] and a significant main effect of CREBαδ KO genotype [*F*_(1_,_12)_ = 102.5, *p* < 0.0001]. Tukey’s *post hoc* group comparisons indicated that the CREBαδ KO mice exhibited a significant baseline reduction (*p* = 0.001) in expression of *cFos* mRNA in the neocortex, and while DOI-evoked a robust and significant upregulation of *cFos* mRNA levels in WT animals (*p* = 0.02) this was completely lost in the CREBαδ KO mice. Neocortical *c-Fos* mRNA levels differed significantly between DOI-treated WT mice and the DOI-treated CREBαδ KO cohort (*p* < 0.0001). In contrast, we observed no significant DOI by CREBαδ KO genotype interaction for *Egr2* mRNA levels within the neocortex (Figure 4J). Further, while we did note a significant main effect of DOI for *Egr2* expression [*F*_(1_,_11)_ = 10.19, *p* = 0.003] we observed no main effect of CREBαδ KO genotype. Collectively, these observations indicate that CREB contributes to the acute DOI-evoked upregulation of *Arc*, *Cebpb*, and *cFos* mRNA within the neocortex, but not to the DOI-induced neocortical increase in *Bdnf1* and *Egr2* mRNA expression. Furthermore, baseline expression of *Cebpb* and *cFos* mRNA, but not *Arc* mRNA is also significantly attenuated in the CREBαδ KO mice. These results demonstrate that the transcription factor CREB contributes to the regulation of a subset of neuronal plasticity-associated genes that are regulated by the hallucinogenic 5-HT_2A_ receptor agonist, DOI in the neocortex.

## Discussion

**FIGURE 5:**
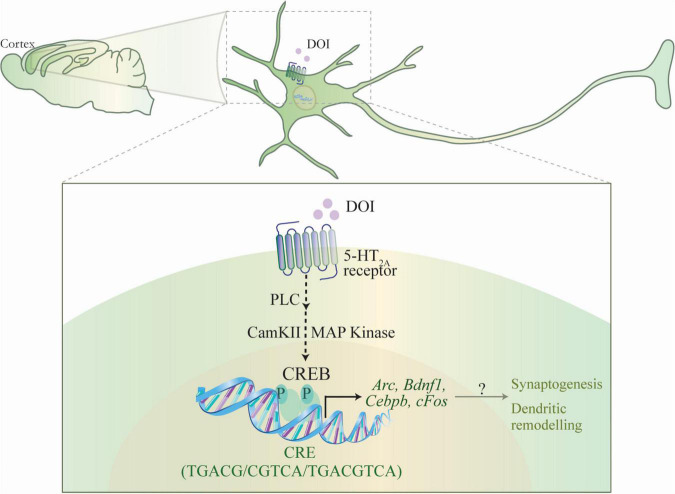
Schematic depicting the putative mechanism for the CREB-dependent regulation of neuronal plasticity-associated gene expression by the hallucinogenic 5-HT_2A_ receptor agonist DOI. DOI, a hallucinogenic agonist of the 5-HT_2A_ receptor is known to evoke a specific transcriptome signature within the neocortex, including the upregulation of the expression of several plasticity-associated genes. The schematic indicates a putative mechanism through which DOI-mediated stimulation of the Gq-coupled 5-HT_2A_ receptor results in the recruitment of the phospholipase C (PLC), MAP kinase and CaMKII signaling pathways, which would further result in the phosphorylation of the transcription factor CREB, thus facilitating the CREB-dependent transcription of plasticity-associated genes, *Arc, Bdnf1*, *Cebpb*, *cFos*. This raises the intriguing possibility that CREB-dependent regulation of gene expression could contribute to the effects of the hallucinogenic 5-HT_2A_ receptor agonist DOI, on neuronal plasticity, synaptogenesis and cell survival.

Here, we show that DOI, a hallucinogenic ligand of the Gq-coupled 5-HT_2A_ receptor, recruits the nuclear transcription factor CREB to influence the expression of a specific subset of neuronal plasticity-associated genes in the rodent neocortex. DOI mediated stimulation of the 5-HT_2A_ receptor in rat cortical neurons recruits the PLC, MAP kinase and CaMKII signaling pathways to rapidly increase the phosphorylation of CREB. 5-HT_2A_ receptor stimulation by DOI, results in the upregulation of *Arc*, *Bdnf1*, *Cebpb*, and *Egr2* mRNA in cortical neurons, with a role for both the MAP kinase and CaMKII signaling pathways. We also noted that while the regulation of *Egr1* is dependent on the MAP kinase pathway, the regulation of *cFos* expression by DOI is dependent on the CaMKII pathway. Further, we show recruitment of pCREB at CRE binding sites within the promoters of a subset of DOI-regulated target genes (*Arc*, *Bdnf1*, *Cebpb*, *cFos*) in the neocortex. In contrast, there are also specific DOI-regulated plasticity-associated genes (*Egr1, Egr2)* wherein we did not observe pCREB enrichment at putative CRE sites in their promoters. These findings collectively indicate that 5-HT_2A_ receptor stimulation enhances pCREB enrichment at the promoters of a subset of DOI regulated target genes, suggestive of an upregulation of neuronal plasticity-associated genes in both a CREB-dependent and independent manner. This is further supported by evidence that the DOI-evoked transcriptional upregulation of specific neuronal plasticity-associated genes (*Arc*, *cFos*, and *Cebpb*) is attenuated in the neocortex of CREBαδ KO mice (Figure 5).

Prior literature indicates that ligands at the 5-HT_2A_ receptor can differentially recruit unique transcriptome fingerprints in the neocortex (González-Maeso et al., 2003), with the hallucinogenic compounds DOI and LSD enhancing the expression of specific transcripts, including *Egr1* and *Egr2* (González-Maeso et al., 2003, 2007). It is interesting that the nature and magnitude of regulation of specific neuronal plasticity-associated genes is comparable to that noted with rapid-action antidepressant treatments such as ECS. Despite the knowledge that hallucinogenic agonists of the 5-HT_2A_ receptor, such as DOI, induce the expression of multiple neuronal plasticity-associated genes, the contribution of specific signaling pathways and transcription factors to the 5-HT_2A_ receptor regulated cortical gene expression remains poorly elucidated. DOI-mediated 5-HT_2A_ receptor stimulation recruits the phospholipase C (PLC)-PKC-MAP kinase cascade and the PLC-CaMKII pathways that are known to also target the transcription factor CREB (Banerjee and Vaidya, 2020; Sharp and Barnes, 2020). The beta-arrestin-receptor complex also acts as a scaffold to activate the Raf-MEK-MAP kinase cascade (Schmid et al., 2008; McCorvy and Roth, 2015). Following 5-HT_2A_ receptor activation by DOI, we observe that both the MAP kinase and CaMKII pathways, contribute to the DOI-evoked transcriptional increase of *Arc*, *Bdnf1*, *Cebpb*, and *Egr2* expression in rat cortical neurons, whilst the CaMKII pathway and the MAP kinase pathway contribute to the DOI-mediated regulation of *cFos* and *Egr1* expression respectively. This highlights the recruitment of distinct signaling pathways by the 5-HT_2A_ receptor agonist, DOI (Banerjee and Vaidya, 2020), several of which converge on the transcription factor CREB (Shaywitz and Greenberg, 1999), placing it as a potential key ‘hub’ regulator of the transcriptional changes that arise in response to DOI treatment. Interestingly, many of the genes we find to be robustly enhanced in expression following DOI treatment in cortical neurons *Arc*, *Bdnf1*, *Cebpb*, *cFos*, *Egr1*, and *Egr2*, are reported to contain putative CRE sites (TGACG/CGTCA/TGACGTCA) (Sheng et al., 1990; Niehof et al., 1997; Ahn et al., 1998; Walters and Blendy, 2001; Fukuchi et al., 2005; Bilbao et al., 2014; Duclot and Kabbaj, 2017; Ly et al., 2020). Our *in vitro* studies clearly indicate a robust induction in pCREB levels at the Ser133 site, with phosphorylation *via* the MAP kinase and CaMKII pathways likely contributing to this induction. Our experiments motivate further characterization of the target sites of CREB phosphorylation (Kornhauser et al., 2002), as certain phosphorylation signatures like Ser142 (Sun et al., 1994) can also serve to evoke an inhibitory effect on CREB mediated transcription.

A previous microarray study from our lab has reported several genes to be upregulated by DOI in the rodent neocortex (Benekareddy et al., 2010). We performed *in silico* analysis on the published array results, and noted that several of the genes upregulated by DOI have CRE elements in their upstream regulatory promoter regions, suggestive of a putative role for CREB in the regulation of multiple DOI-evoked transcripts. DOI-evoked a robust increase in the transcript expression of several CRE-containing genes, namely *Arc*, *Atf3*, *Atf4*, *Bdnf1*, *Cebpb*, *Cebpd*, *Egr1*, *Egr2*, *Egr3*, *Egr4*, *cFos*, *JunB*, and *Nfkbia* in the rat neocortex. Interestingly, we observed pCREB enrichment at the promoters of specific DOI-regulated genes. The expression of *Arc*, *Bdnf1*, *Cebpb*, and *cFos* genes but not *Egr1* and *Egr2* expression was accompanied by a significant enrichment of pCREB at their promoters in the neocortex of DOI-treated animals. In this regard, it is interesting to note that the genes, *Egr1* and *Egr2* which are part of the reported DOI-evoked hallucinogenic fingerprint (González-Maeso et al., 2003) do not appear to show pCREB enrichment at their promoters. While we have restricted our analysis of pCREB recruitment to CRE sites close to the promoter region, we cannot preclude the possibility of CREB binding at remote CRE sites either in distal enhancer regions, or within introns as we have not scanned pCREB enrichment at these loci. It is also important to keep in mind that the differential regulation of target genes by CREB may further be influenced by CRE sequence composition, location of the CRE and distance from the transcription start site (Mayr and Montminy, 2001; Altarejos and Montminy, 2011; Davis et al., 2020). Thus, the presence of a CRE site does not necessarily predict the recruitment of pCREB, and other transcription factors besides CREB are also likely to be recruited by DOI and by neuronal activity-dependent mechanisms, to evoke transcriptional increase of target genes. Our findings provide impetus for a broader genome-wide analysis of pCREB enrichment to get a sense of the span of pCREB-mediated regulation of transcription by DOI, and an understanding of recruitment of pCREB at both canonical and non-canonical CREs.

It is also of interest to note that the target genes regulated by DOI are known to exhibit substantial signaling crosstalk, and could also exert further feedback effects on CREB-dependent transcriptional regulation (Wu et al., 2001; Deisseroth and Tsien, 2002; Wiegert and Bading, 2011; Belgacem and Borodinsky, 2017). *Bdnf*, a DOI-regulated target gene, also contributes to the transcriptional regulation of *Arc* mRNA expression (Bramham et al., 2008; Benekareddy et al., 2012), and could further *via* regulation of the MAP kinase cascade impinge on pCREB mediated gene regulation (Finkbeiner et al., 1997). This raises the intriguing possibility that reciprocal interactions between BDNF and CREB could serve to amplify the regulation of several neuronal plasticity-associated genes (Finkbeiner et al., 1997; Shieh and Ghosh, 1999; Nair and Vaidya, 2006). These include the transcriptional regulation of *Arc* and *c-Fos* which are reported to play a significant role in coupling experience-dependent transcriptional regulation to synaptic plasticity (Duman et al., 2005; Bramham et al., 2008; Flavell and Greenberg, 2008; Shepherd and Bear, 2011; Minatohara et al., 2015). Fos may also function at enhancer elements to coordinate global activity-dependent gene transcription (Malik et al., 2014; Joo et al., 2016). Further, following DOI-evoked stimulation of the 5-HT_2A_ receptor, membrane depolarization and enhanced firing could also recruit additional activity-dependent mechanisms that drive this transcriptional program. This then suggests that stimulation of the 5-HT_2A_ receptor, sets into play a coordinated transcriptional program that involves crosstalk of diverse signaling cascades and transcription factors that drive the expression of several neuronal plasticity-associated genes (*Arc, Bdnf1, cFos*), with CREB being amongst the key hub transcriptional factors that contributes to an important component of the DOI-evoked gene regulation pattern.

We have capitalized on the use of the CREBαδ KO mouse line, which is deficient for the α and δ CREB isoforms leading to a robust reduction in CREB binding to consensus CRE target sites (Walters and Blendy, 2001), to evaluate the contribution of CREB to the effects of DOI on neuronal plasticity-associated gene expression. It is important to note that the CREBαδ KO mice do not exhibit alterations in either the baseline expression of the 5-HT_2A_ receptor, or the stereotypical HTR behavior evoked by the 5-HT_2A_ receptor agonist DOI, that is known to be dependent on the cortical 5-HT_2A_ receptor. This would suggest that alterations in DOI-evoked gene expression in CREBαδ KO mice are unlikely to arise due to a change at the level of 5-HT_2A_ receptor expression and coupling given that the CREBαδ KO mice exhibit a robust DOI-evoked HTR response no different from WT controls and show no change in 5-HT_2A_ receptor expression. Interestingly, the DOI-evoked induction in cortical *Arc* mRNA expression was lost in CREBαδ KO mice. Similar to this observation, the DOI-evoked increase in *Cebpb* and *cFos* mRNA levels were also abolished in the neocortex of CREBαδ KO mice. Further, in CREB deficient mice the baseline expression of *Cebpb* and *cFos*, but not *Arc* mRNA was also reduced, thus supporting a role for CREB in regulation of basal expression of *Cebpb* and *cFos*. The DOI-evoked induction in *Egr2* mRNA was not altered in the neocortex of CREBαδ KO mice, which is consistent with the evidence that pCREB was not found to be enriched at the *Egr2* promoter following DOI treatment. The DOI-evoked increase in *Bdnf1* transcript variant levels was also not significantly attenuated in CREBαδ KO mice, and further we did not observe a change in basal *Bdnf1* transcript expression either. We have focused on *Bdnf1* which has been reported to contain CRE elements at its promoter, and exhibit CREB-mediated regulation in other contexts (Tabuchi et al., 2002; Esvald et al., 2020). *BdnfIII* and *BdnfIV* promoters are also known to contain CRE elements, as is the *Bdnf* coding exon, and it will be important to systematically address the contribution of CREB to the DOI-mediated regulation of multiple *Bdnf* transcript variants (Tao et al., 1998; Esvald et al., 2020). Indeed, prior reports clearly indicate several conditions in which CREB-dependent regulation of *Bdnf* gene expression contributes to neuroplasticity, in particular in the context of neuronal activity-dependent transcriptional coupling that drives structural and synaptic plasticity (Shieh and Ghosh, 1999; Vogt et al., 2014; Yan et al., 2016; Rafa-Zabłocka et al., 2018). While the CREB deficient mice provide a valuable tool to address the contribution of CREB to the DOI-mediated regulation of plasticity-associated gene expression, they come with a caveat of a constitutive, developmental onset loss-of-function of CREB which could in turn disrupt several key signaling pathways. In addition, we have not extensively profiled the consequence of loss of CREBαδ subunits on the composition of the transcription factors that now occupy CRE sites in the absence of CREBαδ, which could itself substantially influence CRE-mediated transcriptional outcomes. Further experiments are warranted to systematically evaluate the contribution of CREB to the effects of DOI on neuronal plasticity-associated genes using approaches that allow for a more targeted strategy of adult onset, neuronal circuit-specific loss of function of CREB.

Our findings highlight a key role for CREB in contributing to the DOI-mediated regulation of specific neuronal plasticity-associated genes in the neocortex. This raises the intriguing possibility that similar to both slow-onset and rapid action antidepressants that recruit CREB to drive transcriptional changes in neurotrophic and plasticity-associated genes (Nibuya et al., 1996; Duman and Nibuya, 1997; Thome et al., 2000; Chen et al., 2001), serotonergic psychedelics that target the 5-HT_2A_ receptor may also recruit CREB to drive a plasticity-associated transcriptional program. These observations encourage further investigation into the role of CREB in regulating the transcription of plasticity-associated genes evoked by hallucinogenic 5-HT_2A_ receptor agonists, thus creating a conducive milieu for the psychoplastogenic actions of serotonergic psychedelics on dendritic plasticity and synaptogenesis in the neocortex.

## Data Availability Statement

The raw data supporting the conclusions of this article will be made available by the authors, without undue reservation.

## Ethics Statement

The animal study was reviewed and approved by the University of Pennsylvania Animal Care and Use Committee. All animal procedures using Sprague-Dawley rats and serotonin_2A_ receptor knockout (5-HT_2A_^–/–^) mice were carried out in accordance with the Committee for Care and Supervision of Experimental Animals (CPCSEA) and approved by the TIFR Institutional Animal Ethics Committee. All experiments with the CREBαδ KO mouse line were carried out in accordance with the NIH guideline for the care and use of laboratory animals.

## Author Contributions

LD, MB, SF, FM, BJ, UG, and TG performed the experiments and analyzed the data. JB and VV designed the experiments and analyzed the data. SF and VV wrote the manuscript. All authors contributed to the article and approved the submitted version.

## Conflict of Interest

The authors declare that the research was conducted in the absence of any commercial or financial relationships that could be construed as a potential conflict of interest.

## Publisher’s Note

All claims expressed in this article are solely those of the authors and do not necessarily represent those of their affiliated organizations, or those of the publisher, the editors and the reviewers. Any product that may be evaluated in this article, or claim that may be made by its manufacturer, is not guaranteed or endorsed by the publisher.

## Footnotes

1
http://www.gene-regulation.com/pub/programs.html

## References

[B1] Aghajanian G. K. Marek G. J. (1999). Serotonin and hallucinogens. *Neuropsychopharmacology* 21 16S–23S. 10.1016/S0893-133X(98)00135-3 10432484

[B2] Ahn S. Olive M. Aggarwal S. Krylov D. Ginty D. D. Vinson C. (1998). A dominant-negative inhibitor of CREB reveals that it is a general mediator of stimulus-dependent transcription of c-fos. *Mol. Cell. Biol.* 18 967–977. 10.1128/MCB.18.2.967 9447994 PMC108809

[B3] Altarejos J. Y. Montminy M. (2011). CREB and the CRTC co-activators: sensors for hormonal and metabolic signals. *Nat. Rev. Mol. Cell Biol.* 12 141–151. 10.1038/nrm3072 21346730 PMC4324555

[B4] Artin H. Zisook S. Ramanathan D. (2021). How do serotonergic psychedelics treat depression: the potential role of neuroplasticity. *World J. Psychiatry* 11 201–214. 10.5498/wjp.v11.i6.201 34168967 PMC8209538

[B5] Banerjee A. A. Vaidya V. A. (2020). Differential signaling signatures evoked by DOI versus lisuride stimulation of the 5-HT2A receptor. *Biochem. Biophys. Res. Commun.* 531 609–614. 10.1016/j.bbrc.2020.08.022 32814630

[B6] Banks M. I. Zahid Z. Jones N. T. Sultan Z. W. Wenthur C. J. (2021). Catalysts for change: the cellular neurobiology of psychedelics. *Mol. Biol. Cell* 32 1135–1144. 10.1091/MBC.E20-05-0340 34043427 PMC8351556

[B7] Belgacem Y. H. Borodinsky L. N. (2017). CREB at the crossroads of activity-dependent regulation of nervous system development and function. *Adv. Exp. Med. Biol.* 1015 19–39. 29080019 10.1007/978-3-319-62817-2_2

[B8] Benekareddy M. Goodfellow N. M. Lambe E. K. Vaidya V. A. (2010). Enhanced function of prefrontal serotonin 5-HT2 receptors in a rat model of psychiatric vulnerability. *J. Neurosci.* 30 12138–12150. 10.1523/JNEUROSCI.3245-10.2010 20826676 PMC4177096

[B9] Benekareddy M. Nair A. R. Dias B. G. Suri D. Autry A. E. Monteggia L. M. (2012). Induction of the plasticity-Associated immediate early gene Arc by stress and hallucinogens: role of brain-derived neurotrophic factor. *Int. J. Neuropsychopharmacol.* 16 405–415. 10.1017/S1461145712000168 22404904

[B10] Berthoux C. Barre A. Bockaert J. Marin P. Bécamel C. (2019). Sustained activation of postsynaptic 5-HT 2A receptors gates plasticity at prefrontal cortex synapses. *Cereb. Cortex* 29 1659–1669. 10.1093/cercor/bhy064 29917056

[B11] Bilbao A. Rieker C. Cannella N. Parlato R. Golda S. Piechota M. (2014). CREB activity in dopamine D1 receptor expressing neurons regulates cocaine-induced behavioral effects. *Front. Behav. Neurosci.* 8:212. 10.3389/fnbeh.2014.00212 24966820 PMC4052973

[B12] Blendy J. A. Kaestner K. H. Schmid W. Gass P. Schutz G. (1996). Targeting of the CREB gene leads to up-regulation of a novel CREB mRNA isoform. *EMBO J.* 15 1098–1106. 8605879 PMC450007

[B13] Bramham C. R. Worley P. F. Moore M. J. Guzowski J. F. (2008). The immediate early gene Arc/Arg3.1: regulation, mechanisms, and function. *J. Neurosci.* 28 11760–11767. 10.1523/JNEUROSCI.3864-08.2008 19005037 PMC2615463

[B14] Canal C. E. Morgan D. (2012). Head-twitch response in rodents induced by the hallucinogen 2,5-dimethoxy-4-iodoamphetamine: a comprehensive history, a re-evaluation of mechanisms, and its utility as a model. *Drug Test. Anal.* 4 556–576. 10.1002/dta.1333 22517680 PMC3722587

[B15] Carlezon W. A. Duman R. S. Nestler E. J. (2005). The many faces of CREB. *Trends Neurosci.* 28 436–445. 10.1016/j.tins.2005.06.005 15982754

[B16] Chen A. C. H. Shirayama Y. Shin K. H. Neve R. L. Duman R. S. (2001). Expression of the cAMP response element binding protein (CREB) in hippocampus produces an antidepressant effect. *Biol. Psychiatry* 49 753–762. 10.1016/S0006-3223(00)01114-8 11331083

[B17] Davis J. E. Insigne K. D. Jones E. M. Hastings Q. A. Boldridge W. C. Kosuri S. (2020). Dissection of c-AMP response element architecture by using genomic and episomal massively parallel reporter assays. *Cell Syst.* 11 75.e7–85.e7. 10.1016/j.cels.2020.05.011 32603702

[B18] De Gregorio D. Aguilar-Valles A. Preller K. H. Heifets B. D. Hibicke M. Mitchell J. (2021). Hallucinogens in mental health: preclinical and clinical Studies on LSD, Psilocybin, MDMA, and ketamine. *J. Neurosci.* 41 891–900. 10.1523/JNEUROSCI.1659-20.2020 33257322 PMC7880300

[B19] de Vos C. M. H. Mason N. L. Kuypers K. P. C. (2021). Psychedelics and neuroplasticity: a systematic review unraveling the biological underpinnings of psychedelics. *Front. Psychiatry* 12:724606. 10.3389/fpsyt.2021.724606 34566723 PMC8461007

[B20] Deisseroth K. Tsien R. W. (2002). Dynamic multiphosphorylation passwords for activity-dependent gene expression. *Neuron* 34 179–182. 10.1016/s0896-6273(02)00664-5 11970860

[B21] Desouza L. A. Sathanoori M. Kapoor R. Rajadhyaksha N. Gonzalez L. E. Kottmann A. H. (2011). Thyroid hormone regulates the expression of the sonic hedgehog signaling pathway in the embryonic and adult Mammalian brain. *Endocrinology* 152 1989–2000. 10.1210/en.2010-1396 21363934 PMC3179409

[B22] Duclot F. Kabbaj M. (2017). The role of early growth response 1 (EGR1) in brain plasticity and neuropsychiatric disorders. *Front. Behav. Neurosci.* 11:35. 10.3389/fnbeh.2017.00035 28321184 PMC5337695

[B23] Duman R. S. Adams D. H. Simen B. B. (2005). “Transcription factors as modulators of stress responsivity,” in *Handbook of Stress and the Brain*, eds Steckler T. Kalin N. H. Reul J. M. H. M. (Amsterdam: Elsevier), 679–698.

[B24] Duman R. S. Nibuya M. V. V. (1997). “A Role for CREB in Antidepressant Action,” in *Antidepressants: New Pharmacological Strategies*, ed. Skolnick P. (Totowa, NJ: Humana Press Inc.), 173–194.

[B25] Esvald E. E. Tuvikene J. Sirp A. Patil S. Bramham C. R. Timmusk T. (2020). CREB family transcription factors are major mediators of BDNF transcriptional autoregulation in cortical neurons. *J. Neurosci.* 40 1405–1426. 10.1523/JNEUROSCI.0367-19.2019 31915257 PMC7044735

[B26] Fanibunda S. E. Deb S. Maniyadath B. Tiwari P. Ghai U. Gupta S. (2019). Serotonin regulates mitochondrial biogenesis and function in rodent cortical neurons *via* the 5-HT2A receptor and SIRT1–PGC-1α axis. *Proc. Natl. Acad. Sci. U.S.A.* 166 11028–11037. 10.1073/pnas.1821332116 31072928 PMC6561197

[B27] Finkbeiner S. Tavazoie S. F. Maloratsky A. Jacobs K. M. Harris K. M. Greenberg M. E. (1997). CREB: a major mediator of neuronal neurotrophin responses. *Neuron* 19 1031–1047. 10.1016/s0896-6273(00)80395-5 9390517

[B28] Flavell S. W. Greenberg M. E. (2008). Signaling mechanisms linking neuronal activity to gene expression and plasticity of the nervous system. *Annu. Rev. Neurosci.* 31 563–590. 10.1146/annurev.neuro.31.060407.125631 18558867 PMC2728073

[B29] Fukuchi M. Tabuchi A. Tsuda M. (2005). Transcriptional regulation of neuronal genes and its effect on neural functions: cumulative mRNA expression of PACAP and BDNF genes controlled by calcium and cAMP signals in neurons. *J. Pharmacol. Sci.* 98 212–218. 10.1254/jphs.fmj05001x4 16006741

[B30] González-Maeso J. Weisstaub N. V. Zhou M. Chan P. Ivic L. Ang R. (2007). Hallucinogens recruit specific cortical 5-HT2A receptor-mediated signaling pathways to affect behavior. *Neuron* 53 439–452. 10.1016/j.neuron.2007.01.008 17270739

[B31] González-Maeso J. Yuen T. Ebersole B. J. Wurmbach E. Lira A. Zhou M. (2003). Transcriptome fingerprints distinguish hallucinogenic and nonhallucinogenic 5-hydroxytryptamine 2A receptor agonist effects in mouse somatosensory cortex. *J. Neurosci.* 23 8836–8843. 10.1523/jneurosci.23-26-08836.2003 14523084 PMC6740401

[B32] Halberstadt A. L. Geyer M. A. (2013). Characterization of the head-twitch response induced by hallucinogens in mice: detection of the behavior based on the dynamics of head movement. *Psychopharmacology* 227 727–739. 10.1007/s00213-013-3006-z 23407781 PMC3866102

[B33] Jefsen O. H. Elfving B. Wegener G. Müller H. K. (2021). Transcriptional regulation in the rat prefrontal cortex and hippocampus after a single administration of psilocybin. *J. Psychopharmacol.* 35 483–493. 10.1177/0269881120959614 33143539

[B34] Joo J.-Y. Schaukowitch K. Farbiak L. Kilaru G. Kim T.-K. (2016). Stimulus-specific combinatorial functionality of neuronal c-fos enhancers. *Nat. Neurosci.* 19 75–83. 10.1038/nn.4170 26595656 PMC4696896

[B35] Koch J. M. Hinze-Selch D. Stingele K. Huchzermeier C. Göder R. Seeck-Hirschner M. (2009). Changes in CREB Phosphorylation and BDNF plasma levels during Psychotherapy of depression. *Psychother. Psychosom.* 78 187–192. 10.1159/000209350 19321972

[B36] Kornhauser J. M. Cowan C. W. Shaywitz A. J. Dolmetsch R. E. Griffith E. C. Hu L. S. (2002). CREB transcriptional activity in neurons is regulated by multiple, calcium-specific phosphorylation events. *Neuron* 34 221–233. 10.1016/s0896-6273(02)00655-4 11970864

[B37] Krishnan V. Nestler E. J. (2008). The molecular neurobiology of depression. *Nature* 455 894–902. 10.1038/nature07455 18923511 PMC2721780

[B38] Krishnan V. Nestler E. J. (2010). Linking molecules to mood: new insight into the biology of depression. *Am. J. Psychiatry* 167 1305–1320. 10.1176/appi.ajp.2009.10030434 20843874 PMC3031089

[B39] Lonze B. E. Ginty D. D. (2002). Function and regulation of CREB family transcription factors in the nervous system. *Neuron* 35 605–623. 10.1016/S0896-6273(02)00828-0 12194863

[B40] López-Giménez J. F. González-Maeso J. (2018). Hallucinogens and serotonin 5-HT2A receptor-mediated signaling pathways. *Curr. Top. Behav. Neurosci.* 36 45–73. 10.1007/7854_2017_478 28677096 PMC5756147

[B41] Ly C. Greb A. C. Cameron L. P. Wong J. M. Barragan E. V. Wilson P. C. (2018). Psychedelics promote structural and functional neural plasticity. *Cell Rep.* 23 3170–3182. 10.1016/j.celrep.2018.05.022 29898390 PMC6082376

[B42] Ly C. Greb A. C. Vargas M. V. Duim W. C. Grodzki A. C. G. Lein P. J. (2020). Transient stimulation with psychoplastogens is sufficient to initiate neuronal growth. *ACS Pharmacol. Transl. Sci.* 4 452–460. 10.1021/acsptsci.0c00065 33860174 PMC8033616

[B43] Madsen M. K. Fisher P. M. Burmester D. Dyssegaard A. Stenbæk D. S. Kristiansen S. (2019). Psychedelic effects of psilocybin correlate with serotonin 2A receptor occupancy and plasma psilocin levels. *Neuropsychopharmacology* 44 1328–1334. 10.1038/S41386-019-0324-9 30685771 PMC6785028

[B44] Malik A. N. Vierbuchen T. Hemberg M. Rubin A. A. Ling E. Couch C. H. (2014). Genome-wide identification and characterization of functional neuronal activity-dependent enhancers. *Nat. Neurosci.* 17 1330–1339. 10.1038/nn.3808 25195102 PMC4297619

[B45] Marek G. J. (2017). “Interactions of hallucinogens with the glutamatergic system: permissive network effects mediated through cortical layer V pyramidal neurons. *Curr. Top. Behav. Neurosci.* 36 107–135. 10.1007/7854_2017_480 28831734

[B46] Martin D. A. Nichols C. D. (2016). Psychedelics recruit multiple cellular types and produce complex transcriptional responses within the brain. *EBioMedicine* 11 262–277. 10.1016/j.ebiom.2016.08.049 27649637 PMC5050000

[B47] Mayr B. Montminy M. (2001). Transcriptional regulation by the phosphorylation-dependent factor creb. *Nat. Rev. Mol. Cell Biol.* 2 599–609. 10.1038/35085068 11483993

[B48] McCorvy J. D. Roth B. L. (2015). Structure and function of serotonin G protein-coupled receptors. *Pharmacol. Ther.* 150 129–142. 10.1016/j.pharmthera.2015.01.009 25601315 PMC4414735

[B49] Minatohara K. Akiyoshi M. Okuno H. (2015). Role of immediate-early genes in synaptic plasticity and neuronal ensembles underlying the memory trace. *Front. Mol. Neurosci.* 8:78. 10.3389/fnmol.2015.00078 26778955 PMC4700275

[B50] Nair A. Vaidya V. A. (2006). Cyclic AMP response element binding protein and brain-derived neurotrophic factor: molecules that modulate our mood? *J. Biosci.* 31 423–434. 10.1007/BF02704114 17006024 PMC4820646

[B51] Nibuya M. Nestler E. J. Duman R. S. (1996). Chronic antidepressant administration increases the expression of cAMP response element binding protein (CREB) in rat hippocampus. *J. Neurosci.* 16 2365–2372. 10.1523/jneurosci.16-07-02365.1996 8601816 PMC6578518

[B52] Nichols C. D. Garcia E. E. Sanders-Bush E. (2003). Dynamic changes in prefrontal cortex gene expression following lysergic acid diethylamide administration. *Brain Res. Mol. Brain Res.* 111 182–188. 10.1016/s0169-328x(03)00029-9 12654518

[B53] Niehof M. Manns M. P. Trautwein C. (1997). CREB controls LAP/C/EBP beta transcription. *Mol. Cell. Biol.* 17 3600–3613. 10.1128/MCB.17.7.3600 9199295 PMC232213

[B54] Nutt D. Erritzoe D. Carhart-Harris R. (2020). Psychedelic Psychiatry’s brave new world. *Cell* 181 24–28. 10.1016/j.cell.2020.03.020 32243793

[B55] Olson D. E. (2018). Psychoplastogens: a promising class of plasticity-promoting neurotherapeutics. *J. Exp. Neurosci.* 12:1179069518800508. 10.1177/1179069518800508 30262987 PMC6149016

[B56] Preller K. H. Burt J. B. Ji J. L. Schleifer C. H. Adkinson B. D. Stämpfli P. (2018). Changes in global and thalamic brain connectivity in LSD-induced altered states of consciousness are attributable to the 5-HT2A receptor. *eLife* 7:e35082. 10.7554/eLife.35082 30355445 PMC6202055

[B57] Rafa-Zabłocka K. Kreiner G. Bagińska M. Nalepa I. (2018). Selective depletion of CREB in serotonergic neurons affects the upregulation of brain-derived neurotrophic factor evoked by chronic fluoxetine treatment. *Front. Neurosci.* 12:637. 10.3389/fnins.2018.00637 30294251 PMC6158386

[B58] Raymond J. R. Mukhin Y. V. Gelasco A. Turner J. Collinsworth G. Gettys T. W. (2001). Multiplicity of mechanisms of serotonin receptor signal transduction. *Pharmacol. Ther.* 92 179–212. 10.1016/s0163-7258(01)00169-3 11916537

[B59] Sakamoto K. Karelina K. Obrietan K. (2011). CREB: a multifaceted regulator of neuronal plasticity and protection. *J. Neurochem.* 116 1–9. 10.1111/j.1471-4159.2010.07080.x 21044077 PMC3575743

[B60] Savalia N. K. Shao L. X. Kwan A. C. (2021). A dendrite-focused framework for understanding the actions of ketamine and psychedelics. *Trends Neurosci.* 44 260–275. 10.1016/j.tins.2020.11.008 33358035 PMC7990695

[B61] Savino A. Nichols C. D. (2021). Lysergic acid diethylamide induces increased signalling entropy in rats’ prefrontal cortex. *J. Neurochem.* [Epub ahead of print]. 10.1111/jnc.15534 34729786 PMC9298798

[B62] Schmid C. L. Raehal K. M. Bohn L. M. (2008). Agonist-directed signaling of the serotonin 2A receptor depends on beta-arrestin-2 interactions *in vivo*. *Proc. Natl. Acad. Sci. U.S.A.* 105 1079–1084. 10.1073/pnas.0708862105 18195357 PMC2242710

[B63] Schmittgen T. D. Livak K. J. (2008). Analyzing real-time PCR data by the comparative C(T) method. *Nat. Protoc.* 3 1101–1108. 10.1038/nprot.2008.73 18546601

[B64] Shao L. X. Liao C. Gregg I. Davoudian P. A. Savalia N. K. Delagarza K. (2021). Psilocybin induces rapid and persistent growth of dendritic spines in frontal cortex *in vivo*. *Neuron* 109 2535.e4–2544.e4. 10.1016/j.neuron.2021.06.008 34228959 PMC8376772

[B65] Sharp T. Barnes N. M. (2020). Central 5-HT receptors and their function; present and future. *Neuropharmacology* 177:108155. 10.1016/j.neuropharm.2020.108155 32522572

[B66] Shaywitz A. J. Greenberg M. E. (1999). CREB: a stimulus-induced transcription factor activated by a diverse array of extracellular signals. *Annu. Rev. Biochem.* 68 821–861. 10.1146/annurev.biochem.68.1.821 10872467

[B67] Sheng M. McFadden G. Greenberg M. E. (1990). Membrane depolarization and calcium induce c-fos transcription *via* phosphorylation of transcription factor CREB. *Neuron* 4 571–582. 10.1016/0896-6273(90)90115-v 2157471

[B68] Shepherd J. D. Bear M. F. (2011). New views of Arc, a master regulator of synaptic plasticity. *Nat. Neurosci.* 14 279–284. 10.1038/nn.2708 21278731 PMC8040377

[B69] Shieh P. B. Ghosh A. (1999). Molecular mechanisms underlying activity-dependent regulation of BDNF expression. *J. Neurobiol.* 41 127–134. 10504200

[B70] Slocum S. T. DiBerto J. F. Roth B. L. (2021). Molecular insights into psychedelic drug action. *J. Neurochem.* [Epub ahead of print]. 10.1111/jnc.15540 34797943

[B71] Sun P. Enslen H. Myung P. S. Maurer R. A. (1994). Differential activation of CREB by Ca2+/calmodulin-dependent protein kinases type II and type IV involves phosphorylation of a site that negatively regulates activity. *Genes Dev.* 8 2527–2539. 10.1101/gad.8.21.2527 7958915

[B72] Tabuchi A. Sakaya H. Kisukeda T. Fushiki H. Tsuda M. (2002). Involvement of an upstream stimulatory factor as well as cAMP-responsive element-binding protein in the activation of brain-derived neurotrophic factor gene promoter I. *J. Biol. Chem.* 277 35920–35931. 10.1074/jbc.M204784200 12114522

[B73] Tao X. Finkbeiner S. Arnold D. B. Shaywitz A. J. Greenberg M. E. (1998). Ca2+ influx regulates BDNF transcription by a CREB family transcription factor-dependent mechanism. *Neuron* 20 709–726. 10.1016/S0896-6273(00)81010-7 9581763

[B74] Thome J. Sakai N. Shin K. H. Steffen C. Zhang Y. J. Impey S. (2000). cAMP response element-mediated gene transcription is upregulated by chronic antidepressant treatment. *J. Neurosci.* 20 4030–4036. 10.1523/jneurosci.20-11-04030.2000 10818138 PMC6772651

[B75] Titeler M. Lyon R. A. Glennon R. A. (1988). Radioligand binding evidence implicates the brain 5-HT2 receptor as a site of action for LSD and phenylisopropylamine hallucinogens. *Psychopharmacology* 94 213–216. 10.1007/BF00176847 3127847

[B76] Vaidya V. A. Marek G. J. Aghajanian G. K. Duman R. S. (1997). 5-HT2A receptor-mediated regulation of brain-derived neurotrophic factor mRNA in the hippocampus and the neocortex. *J. Neurosci.* 17 2785–2795. 9092600 10.1523/JNEUROSCI.17-08-02785.1997 PMC6573109

[B77] Vargas M. V. Meyer R. Avanes A. A. Rus M. Olson D. E. (2021). Psychedelics and other psychoplastogens for treating mental illness. *Front. Psychiatry* 12:727117. 10.3389/fpsyt.2021.727117 34671279 PMC8520991

[B78] Vogt M. A. Inta D. Luoni A. Elkin H. Pfeiffer N. Riva M. A. (2014). Inducible forebrain-specific ablation of the transcription factor Creb during adulthood induces anxiety but no spatial/contextual learning deficits. *Front. Behav. Neurosci.* 8:407. 10.3389/FNBEH.2014.00407 25505876 PMC4245921

[B79] Vollenweider F. X. Preller K. H. (2020). Psychedelic drugs: neurobiology and potential for treatment of psychiatric disorders. *Nat. Rev. Neurosci.* 21 611–624. 10.1038/S41583-020-0367-2 32929261

[B80] Vollenweider F. X. Vollenweider-Scherpenhuyzen M. F. I. Bäbler A. Vogel H. Hell D. (1998). Psilocybin induces schizophrenia-like psychosis in humans *via* a serotonin-2 agonist action. *Neuroreport* 9 3897–3902. 10.1097/00001756-199812010-00024 9875725

[B81] Walters C. L. Blendy J. A. (2001). Different requirements for cAMP response element binding protein in positive and negative reinforcing properties of drugs of abuse. *J. Neurosci.* 21 9438–9444. 11717377 10.1523/JNEUROSCI.21-23-09438.2001 PMC6763933

[B82] Weisstaub N. V. Zhou M. Lira A. Lambe E. González-Maeso J. Hornung J.-P. (2006). Cortical 5-HT2A receptor signaling modulates anxiety-like behaviors in mice. *Science* 313 536–540. 10.1126/science.1123432 16873667

[B83] Wiegert J. S. Bading H. (2011). Activity-dependent calcium signaling and ERK-MAP kinases in neurons: a link to structural plasticity of the nucleus and gene transcription regulation. *Cell Calcium* 49 296–305. 10.1016/j.ceca.2010.11.009 21163523

[B84] Wu G. Y. Deisseroth K. Tsien R. W. (2001). Activity-dependent CREB phosphorylation: convergence of a fast, sensitive calmodulin kinase pathway and a slow, less sensitive mitogen-activated protein kinase pathway. *Proc. Natl. Acad. Sci. U.S.A.* 98 2808–2813. 10.1073/pnas.051634198 11226322 PMC30221

[B85] Yan X. Liu J. Ye Z. Huang J. He F. Xiao W. (2016). CaMKII-mediated CREB phosphorylation is involved in Ca2+-Induced BDNF mRNA transcription and neurite outgrowth promoted by electrical stimulation. *PLoS One* 11:e0162784. 10.1371/journal.pone.0162784 27611779 PMC5017744

