# Supplementary material for: The Hallucinogenic Serotonin2A Receptor Agonist, 2,5-Dimethoxy-4-Iodoamphetamine, Promotes cAMP Response Element Binding Protein-Dependent Gene Expression of Specific Plasticity-Associated Genes in the Rodent Neocortex
Source: Front Mol Neurosci. 2021 Dec 24;14:790213. doi: 10.3389/fnmol.2021.790213 (PMC8739224; doi:10.3389/fnmol.2021.790213)
Supplement: Supplementary file 1 [file Data_Sheet_1.pdf]

Supplementary Material

1. Supplementary Tables

List of Primers

Supplementary Table 1: Primer sequences used for quantitative PCR (qPCR) analysis of rat/mouse cDNA

Gene name	Primer	Sequence (5'-3')
**RAT**		
Arc	Forward	GCCCCCAGCAGTGATTCATA
	Reverse	GACTCAGCCCCTCTGGGAC
Bdnf1	Forward	AGGGCAGTTGGACAGTCATTGGTA
	Reverse	TTCAACTCTCATCCACCTTGGCGA
Cebpb	Forward	CAAGCTGAGCGACGAGTACA
	Reverse	GACAGCTGCTCCACCTTCTT
cFos	Forward	TGAAGACCATGTCAGGCGG
	Reverse	TTCCCTTCGGATTCTCCGT
Egr1	Forward	AGCAGCGCTTTCAATCCTCA
	Reverse	GAAAAGGACTCTGTGGTCAGGTG
Egr2	Forward	CTGCCTGACAGCCTCTACCC
	Reverse	ATGCCATCTCCAGCCACTCC
Hprt	Forward	GCAGACTTTGCTTTCCTTGG
	Reverse	GTCTGGCCTGTATCCAACACT
Atf3	Forward	GATTCGCCATCCAGAACAAG
	Reverse	TCAGTTCGGCATTCACACTC

Atf4	Forward	AGAATGGCTGGCTATGGATGGGTT
	Reverse	CATGTGTCATCCAACGTGGCCAAA
Cebpd	Forward	CTACAGTTTCTTGGGCACTGG
	Reverse	GCGCAGTCTCTTCCTCTTATCT
Egr3	Forward	ACTCGGTAGCCCATTACACTCAG
	Reverse	GTAGGTCACGGTCTTGTTGCC
Egr4	Forward	GAGCAGGCGATACCTTAGGAC
	Reverse	CCAAAGGCTTGCTGGACTTG
JunB	Forward	TCTCAGCCTTGAGTGTCTTCACCT
	Reverse	ATGGAAGACCAGGAGCGCATCAAA
Nfkbia	Forward	GGACGAGGATTACGAGCAGA
	Reverse	CCCTTCACCTGACCAATCAC
Vegfa	Forward	TCCAATTGAGACCCTGGTGGACAT
	Reverse	TCTCCTATGTGCTGGCTTTGGTGA
**MOUSE**		
Arc	Forward	GCCCCCAGCAGTGATTCATA
	Reverse	GACTCAGCCCCTCTGGGAC
Bdnf1	Forward	CCTGCATCTGTTGGGGAGAC
	Reverse	GCCTTGTCCGTGGACGTTTA
Cebpb	Forward	TGATGCAATCCGGATCAAACGTGG
	Reverse	TTTAAGTGATTACTCAGGGCCCGGCT
cFos	Forward	ATCCTTGGAGCCAGTCAAGAGCAT
	Reverse	TCCCAGTCTGCTGCATAGAAGGAA

Egr1	Forward	TGAGCTTACCCGCCATATCC
	Reverse	CGTTCATCACTCCTGGCAAAC
Egr2	Forward	TCCTTTGATCGAGGACAGC
	Reverse	TGGCCAGAGAAACCTCCATCACT
Htr2a	Forward	CGTGTCCATGTTAACCATCC
	Reverse	TCAGGAAGGCTTTGGTTCTG
Htr2c	Forward	TGAAGCAATAATGGTGAACCTG
	Reverse	GAATTGAAACAAGCGTCCAC
Hprt	Forward	AGGAGTCCTGTTGATGTTGCCAGT
	Reverse	GGGACGCAGCAACTGACATTTCTA

Supplementary Table 2: pCREB ChIP-qPCR primer sequences

**Gene name**	**Primer**	**Sequence (5'-3')**
Arc	Forward	CCGCCTCAGAGGAGTTCTTA
	Reverse	GTGCAACCCTTTCAGCTCTC
Bdnf1	Forward	TGATCATCACTCACGACCACG
	Reverse	CAGCCTCTCTGAGCCAGTTACG
Cebpb	Forward	AGATGTCATTTCTCCAGCTC
	Reverse	ACTCTTCTGCCACTCCCAGC
cFos	Forward	TCTCTGTTCCGCTCATGACGTAGT
	Reverse	CGGCTCTATCCAGTCTTCTCAGTT
Egr1	Forward	AGGCTTTCCAGGAGCCTGAG
	Reverse	GCCCTAATATGGAAGGACCG

Egr2	Forward	TATGCAAATTGGCCATGTGACCGGC
	Reverse	AATCGCTGCTCTCTCAGACGGAAA

2. Supplementary Figures

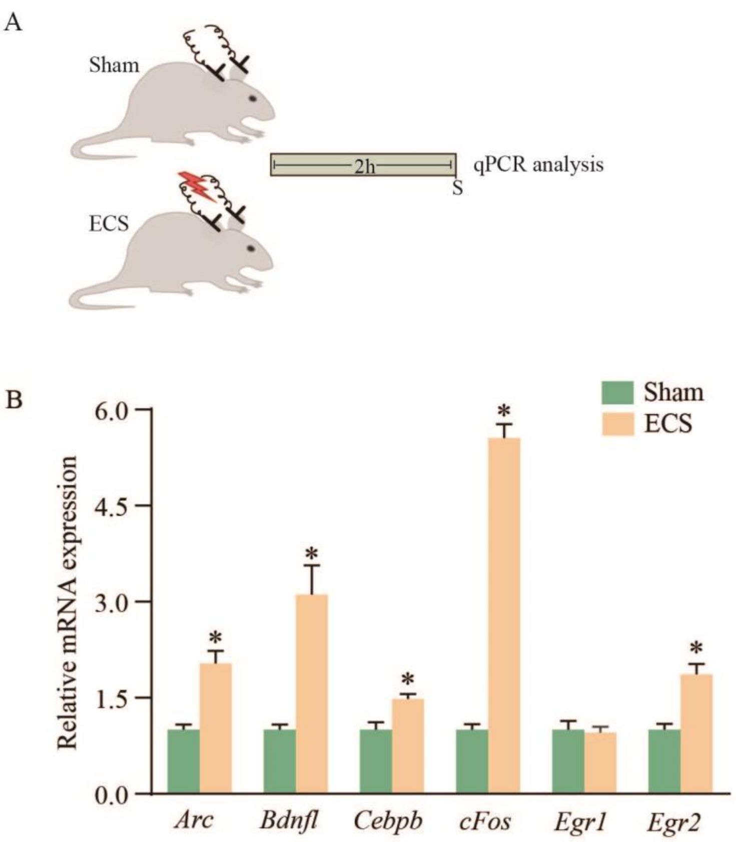

Supplementary Figure 1. *Acute electroconvulsive seizure (ECS) treatment regulates neuronal plasticity-associated gene expression in the rat neocortex*

(A) Shown is a schematic of the experimental design for acute ECS treatment. (B) The bar graph indicates the fold change in mRNA expression of specific neuronal plasticity-associated genes in the neocortex of sham and ECS treated rats represented as fold change of sham ± SEM (n = 6/sham group, n = 10/ECS group, *$p < 0.05$ as compared to sham, unpaired Students *t*-test).
